# Design of FOPID Controller for Pneumatic Control Valve Based on Improved BBO Algorithm

**DOI:** 10.3390/s22176706

**Published:** 2022-09-05

**Authors:** Min Zhu, Zihao Xu, Zhaoyu Zang, Xueping Dong

**Affiliations:** 1 School of Electrical Engineering and Automation, Hefei University of Technology, Hefei 230009, China; 2 Engineering Technology Research Center of Industrial Automation, Hefei 230009, China

**Keywords:** pneumatic control valve, fractional-order PID controller, improved biogeography-based optimization algorithm

## Abstract

Aiming at the problems of nonlinearity and inaccuracy in the model of the pneumatic control valve position in the industrial control process, a valve position control method based on a fractional-order PID controller is proposed. The working principle of the pneumatic control valve is analyzed, and its mathematical model is established. In order to improve the accuracy of the model, an improved biogeography-based optimization algorithm is proposed to tune the parameters of the fractional-order PID controller in view of the wide range and high complexity of the fractional-order PID controller. The initialization of the chaotic graph, the adjustment of the migration model, and the improvement of the migration operator and the mutation operator are introduced to improve the algorithm optimization ability, which is used for the model identification of the control valve control system. The simulation and experimental results clearly show that, compared with the integer-order PID controller, the designed fractional-order PID controller has faster response speed and control accuracy, which can better meet the requirements of pneumatic control valve position control.

## 1. Introduction

As the key terminal equipment in the automation system, the pneumatic control valve is widely used in industrial control fields such as metal smelting, petrochemical, nuclear power, and sewage treatment [1,2]. Due to its inherent properties such as sealing performance, friction force, and flow characteristic curve, the pneumatic control valve inevitably has nonlinear characteristics such as hysteresis and dead zone [3]. In the industrial production process, if the valve position is not properly controlled and the vibration is too large, it will increase the wear of the valve stem, and, in severe cases, it will cause surge and reduce the life of the regulating valve. If the adjustment time is too long, it is not conducive to production efficiency. Pneumatic control valves not only need to reach the specified valve position quickly and smoothly, but also need to have high accuracy.

For the modeling of the pneumatic actuator of the pneumatic control valve, different objects have been investigated, including the modeling and analysis of soft pneumatic actuator based on a soft robot gripper [4], dynamic modeling of bidirectional pneumatic actuator based on the dynamic balance equation [5], and modeling of soft pneumatic actuators with different orientation angles using echo state networks for irregular time series data [6], which are of great help in modeling pneumatic control valves.

Many scholars have also conducted studies on the valve position control of pneumatic control valves. Plestan et al. [7] designed a new adaptive sliding mode controller that ensures that the gain is not overestimated and reduces chattering during valve position control. Haslinda et al. [8] applied predictive control to the pneumatic control valve. Although the nonlinear factor interference of the control valve was solved in a certain sense, the problems of poor robustness and low stability still existed. Guo et al. [9] designed an active disturbance rejection controller according to the characteristics of the valve cylinder servo system. The co-simulation of AMESim and MATLAB verified that the controller has the advantages of strong anti-disturbance and high precision. In addition, fuzzy neural network-PID [10], PID-IMC (internal model control) [11], Expert-PID [12], etc. have been proposed for regulating valve position control, thus improving the control accuracy and response speed of the pneumatic control valve, control accuracy, and responsiveness. At present, most of the control strategies in engineering are still mainly integer-order PID or other control strategies based on integer-order PID, while traditional integer-order PID struggles to meet the increasing control demand.

In the existing literature, few researchers have applied the fractional-order control theory to the valve position control of pneumatic control valves. Because the fractional calculus operation has memory characteristics, compared with the integer-order PID, the differential order and the integral order are introduced. Second, the flexibility of controller design is increased, and the combination of fractional-order calculation and controller parameter tuning is one of the current research hotspots [13,14]. The main methods of fractional-order PID controller parameter tuning include intelligent optimization method [15], phase angle margin and amplitude margin method [16], dominant pole method [17], and transfer function design method based on ideal bode [18]. Some scholars have introduced intelligent optimization algorithms to adjust fractional-order PID parameters, showing good results [19,20,21]. For example, the biogeography-based optimization algorithm, as an intelligent optimization algorithm, has been proven to have fast convergence and high accuracy.

For some current optimization algorithms applied to valve positioner opening control, there are still too many iterations, and the problem of jumping out of the local optimal ability is poor. In order to effectively realize the valve position control of the regulating valve, this paper proposes an improved biogeography-based optimization algorithm, which improves the optimization ability by introducing chaotic mapping initialization, adjusting the migration model, and improving the migration operator and mutation operator. The model is not accurate enough because it does not consider the air pressure fluctuation, system viscosity, and dead zone. Although previous researchers have conducted some forward-looking work on the pneumatic control valve, the current pneumatic control valve still has the problems of inaccurate valve position control, a considerable amount of overshoot, and long adjustment time. Therefore, this paper adopts an improved biogeography-based optimization algorithm to fit the open-loop response curve of the control system, as well as derives a new pneumatic control valve model. In addition, the fractional-order PID control method is applied to the valve position control of the pneumatic control valve, and the parameters of the fractional-order PID controller are adjusted using the proposed improved algorithm. Lastly, the effectiveness of the proposed control valve position control method is proven by simulation and experiment.

## 2. Establishment of Mathematical Model of Pneumatic Control Valve

### 2.1. Pneumatic Control Valve Structure and Working Principle

**Figure 1:**
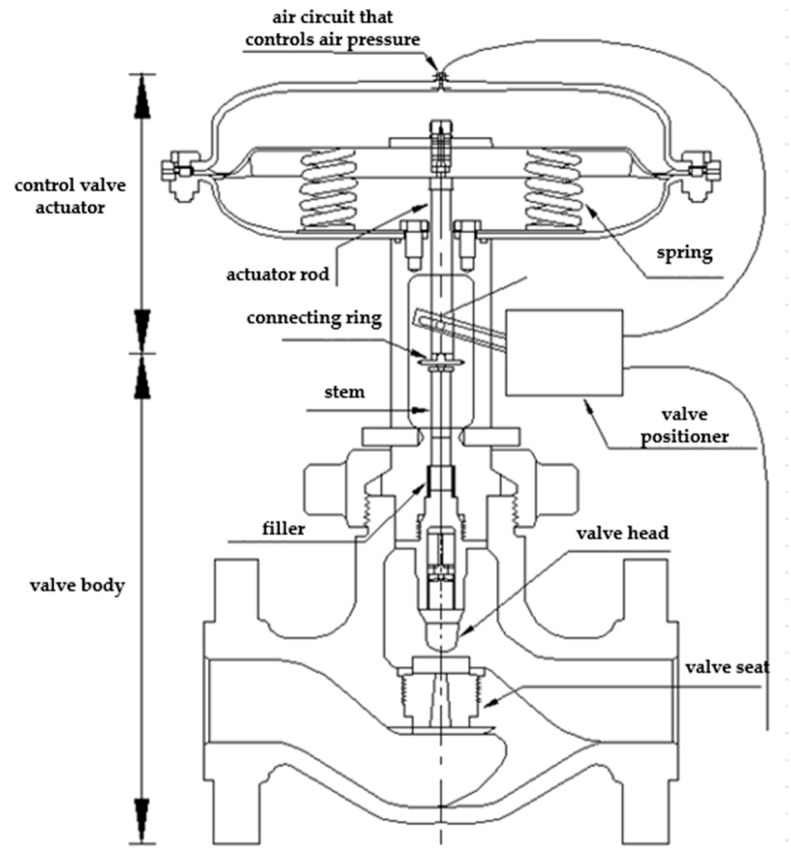
Structural diagram of pneumatic control valve.

**Figure 2:**
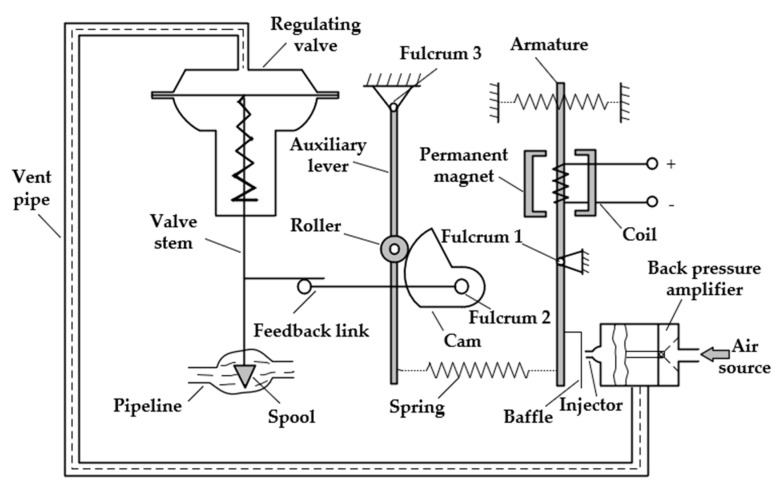
Internal structural diagram of the valve positioner.

**Figure 3:**
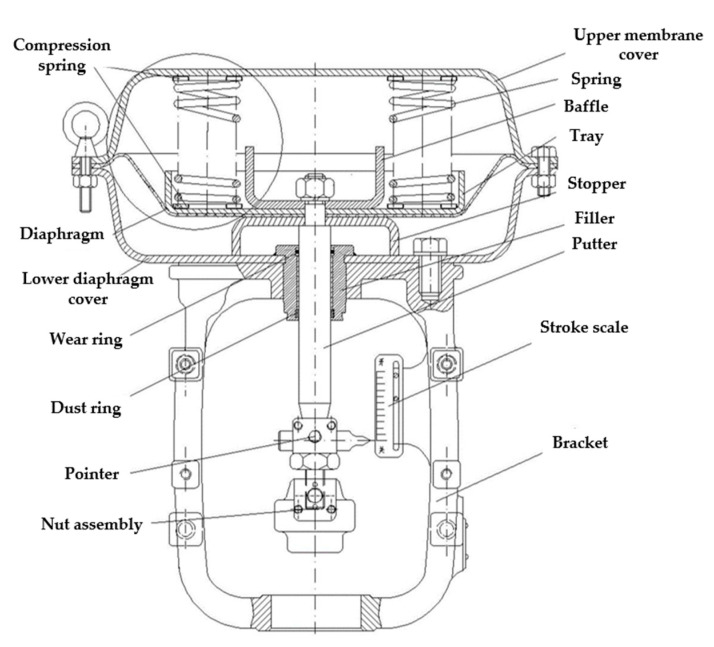
Structural diagram of the valve actuator.

**Figure 4:**
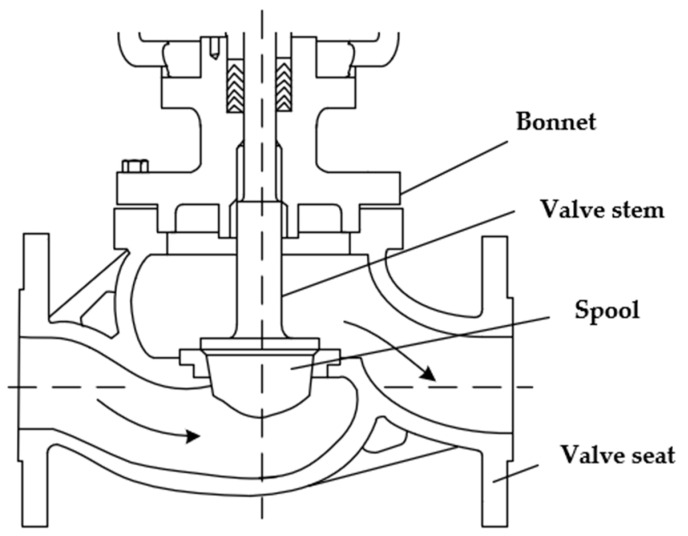
Structural diagram of the control valve body.

The pneumatic control valve is mainly composed of three parts: valve positioner, valve actuator, and valve body. Schematic diagrams of its structure are shown in Figure 1, Figure 2, Figure 3 and Figure 4. The valve positioner is the “brain” of the regulating valve, which calculates the control signal and sends the air pressure signal to the actuator to correct the valve position. As an actuator, the valve actuator adjusts the valve position under the signal of air pressure until the air chamber reaches a balanced state. The valve body is composed of a throttling part through the valve core and the valve seat, so as to realize the corresponding relationship between the flow rate and the valve position.

The working principle of the pneumatic control valve is that the valve positioner receives the valve position setting signal, and the controller processes the valve position setting signal and the collected valve position feedback signal in real time. The output signal of the valve positioner cannot directly drive the valve actuator. The electric/pneumatic conversion unit in the positioner converts the air pressure signal into the air pressure signal, and the converted air pressure signal is amplified by the pneumatic amplifier and then enters the chamber through the control air pressure air path, changes the chamber air pressure, pushes the film to generate thrust, and pushes the valve stem in the valve actuator. Ideally, when the valve position feedback signal and the preset signal are equal, the diaphragm in the actuator chamber is in a balanced state, and the valve position reaches the specified position at this time.

### 2.2. Model Establishment of Pneumatic Control Valve

**Figure 5:**
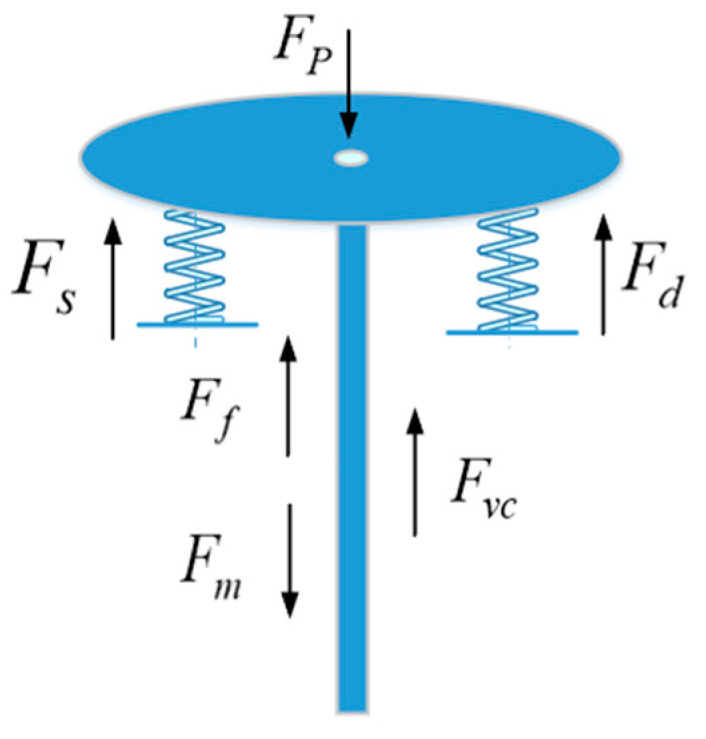
Stress analysis of valve stem.

The pneumatic membrane actuator is divided into a valve actuator mechanism and a regulating mechanism. The working process of the valve actuator mechanism is divided into three stages; the input air pressure is converted into the air pressure in the air chamber, the air chamber air pressure is converted into thrust, and the thrust is converted into valve stem displacement. The last two links can be treated as linear links. The differential equation of the valve actuator mathematical model is approximated as

(1) $$RC\frac{K_{r}}{A_{e}}\frac{dP_{2}}{dt}+P_{2}=P_{1},$$

where $P_{1}$ is the input air pressure, $P_{2}$ is the chamber air pressure, $K_{r}$ is the spring stiffness, $A_{e}$ is the effective area of the diaphragm in the chamber, R is the air resistance of the air path ($R=\beta_{1}\cdot \frac{l}{s}$, where $\beta_{1}$ is the gas resistance coefficient of the gas path, l is the length of the gas path, and s is the cross-sectional area of the gas path), and C is the air capacity of the air path ($C=\beta_{2}V$, where $\beta_{2}$ is the gas capacity coefficient of the gas path, and V is the volume of the gas cavity). The stress analysis diagram of the valve stem is shown in Figure 5.

The rod of the adjustment mechanism is driven by the spring structure, and a force analysis of the valve rod is carried out. When the sum of the pressure of the chamber gas on the chamber film and the gravity of the valve rod is greater than the sum of the friction force and the spring force of the valve rod, the valve rod will slide down. According to Newton’s second law, we have

(2) $$ma=F_{m}+F_{p}-F_{f}-F_{d}-F_{s}-F_{vc},$$

where $F_{m}$ is the gravity of the valve stem, i.e., the gas chamber membrane above the valve stem, the pressure $F_{p}$ is generated by the gas in the gas chamber acting on the membrane, the static friction $F_{f}$ is generated between the valve stem and the packing, the preload $F_{d}$ of the spring is generated on the valve stem when it leaves the factory, the reaction force $F_{s}$ of the spring is generated on the valve stem when it is compressed, the reaction force $F_{vc}$ of the medium flowing through the valve body is generated on the valve stem, ignoring the spring preload and the reaction force. Unfolding Equation (2) yields

(3) $$m\ddot{x}=mg+pA_{e}-K_{b}\dot{x}-K_{f}x,$$

where m is the mass of the valve stem, g is the acceleration due to gravity, p is the chamber pressure, $K_{b}$ is the Coulomb friction coefficient of the valve stem, and $K_{f}$ is the spring stiffness coefficient.

The valve positioner is mainly composed of a torque motor, nozzle baffle, and pneumatic amplifier. The torque motor includes a coil circuit and a magnetic unit, and the relationship between its output torque and the deflection angle of the baffle is simplified as follows:

(4) $$T_{d}=K_{t}\Delta i+K_{m}\theta ,$$

(5) $$K_{t}=2N_{c}\phi_{g}\left(\frac{a}{g}\right)\xi ,$$

(6) $$K_{m}=4\phi^{2}R_{g}{\left(\frac{a}{g}\right)}^{2}\xi ,$$

where $K_{t}$ is the electromagnetic torque coefficient of the torque motor, $K_{m}$ is the magnetic spring stiffness of the torque motor, Δi is the input current, $N_{c}$ is the number of turns of the coil, ϕg is the initial zero magnetic flux, a is the length of the moment arm, g is the air gap width of the iron piece in the middle position, and ξ is the influence coefficient of the reluctance in the magnetic circuit on the torque motor.

The distance between the orifice of the nozzle baffle and the baffle is determined by the output angle of the torque motor. The flow characteristics of the nozzle baffle are simplified as follows:

(7) $$Q_{L}=\sqrt{\frac{1}{\rho }P_{s}}\left(\frac{\pi C_{q}D_{0}{}^{2}}{4}-\pi C_{q}D_{f}x_{f}\right),$$

where ρ is the fluid density, $P_{s}$ is the gas source air pressure, $Q_{L}$ is the flow rate of the nozzle baffle, $C_{q}$ is the flow coefficient (generally 0.6–0.8), $D_{0}$ is the diameter of the orifice, $D_{f}$ is the nozzle diameter, and $x_{f}$ is the offset of baffle to equilibrium state.

The pneumatic amplifier amplifies the output air pressure of the nozzle baffle, such that the gas enters the film air chamber to drive the valve stem to move. The working process of charging and exhausting can be equivalent to the flow characteristics of small holes. The flow equation at the valve opening is as follows:

(8) $$\left\{ \begin{array}{ll} q_{m}{}^{*}=0.04\frac{P_{s}}{\sqrt{T_{s}}} & \frac{P}{P_{s}}<b\;\\ q_{m}=q_{m}{}^{*}\sqrt{1-{\left(\frac{\frac{P}{P_{s}}-b}{1-b}\right)}^{2}} & \;b\leq \frac{P}{P_{s}}\leq 1 \end{array}\right.,$$

where $q_{m}{}^{*}$ and $q_{m}$ represent the gas mass flow in the case of sonic flow and subsonic flow, respectively, $T_{s}$ is the air temperature upstream of the orifice, b is the critical pressure ratio, and P is the downstream pressure of the orifice.

## 3. Biogeography-Based Optimization Algorithms and Improvements

### 3.1. Overview of Standard Biogeography-Based Optimization Algorithms

Since the genetic algorithm was proposed in 1963, more than half a century later, people are continuously proposing various meta-heuristic algorithms through biological behavior, natural principles, and even social phenomena and applying them to solve various problems in social life.

Professor Dan Simon formally proposed the biogeography-based optimization algorithm in IEEE Transsctions on Evolutionary Computition in 2008 [22]. Because the biogeography-based optimization algorithm is simple, is easy to implement, and has few parameters, it has attracted extensive attention from scholars all over the world. The method uses biogeographic principles for mathematical modeling and simulates species movement and information exchange between island habitats, resulting in a biogeographic optimization process.

### 3.2. Description of Biogeography-Based Optimization Algorithm Operators

The standard biogeography-based optimization algorithm mainly includes three kinds of operators: migration operator, mutation operator, and clearing operator.

The role of the migration operator is mainly to exchange information between the selected island habitats and the island habitats selected by the roulette wheel in the remaining island habitats.

The role of the mutation operator is to perform random mutation within the upper and lower bounds of a dimension on the selected island habitat.

The role of the removal operator is to remove the duplicate species in the island habitat after a series of operations on the species in the island habitat (retaining one of the duplicate species, while the others are randomly mutated within the upper and lower bounds).

### 3.3. Improvement of Biogeography-Based Optimization Algorithm

#### 3.3.1. Chaos Initialization

The chaotic system is random and ergodic, and it can initialize all points in the target area through iterative coverage, making the species in the island habitat more random and uncertain. To this end, a chaotic map sequence is introduced. The chaotic system mapping equation is as follows:

(9) Z(1,:)=rand(1,OPTIONS.numVar),

(10) Z(i,:)=μZ(i−1,:). ×(1−Z(i−1,:)),i=2,3,…,OPTIONS.popsize,

where OPTIONS.numVar is the number of genes in each population member, OPTIONS.popsize is the total population size, and μ is the chaotic variable. When μ=4, the model is in a completely chaotic state.

The chaotic vector is inversely transformed to the original space by Equation (11).

(11) $$\begin{array}{l} chrom=MinParValue+(MaxParValue-MinParValue+1)\times (Z(popindex,:))\\ popindex=1,2,\ldots ,OPTIONS.numVar \end{array}$$

where chrom is the component matrix of the popindex-th island habitat.

#### 3.3.2. Improvements to the Migration Model

Simon adopted the standard biogeography-based optimization algorithm’s migration model as a linear model, suggesting that, with the increase in the number of species, the in-migration rate would show a linear decreasing trend, while the in-migration rate would show a linear migration trend. However, this model is relatively simple, as it can only briefly describe the migration law of species between island habitats, but cannot fully and effectively describe the real migration law. In this paper, a hyperbolic tangent model is used.

(12) $$\begin{array}{l} \lambda_{n}=\frac{I}{2}(-\frac{\alpha^{n-\frac{p}{2}}-\alpha^{-n+\frac{p}{2}}}{\alpha^{n-\frac{p}{2}}+\alpha^{-n+\frac{p}{2}}}+1)\\ \mu_{n}=\frac{E}{2}(\frac{\alpha^{n-\frac{p}{2}}-\alpha^{-n+\frac{p}{2}}}{\alpha^{n-\frac{p}{2}}+\alpha^{-n+\frac{p}{2}}}+1) \end{array}$$

where $\lambda_{n}$ and $\mu_{n}$ are the in-migration rate and the out-migration rate, respectively; when the number of species in the island habitat is *n*, I and E are the highest in- and out-migration rates in island habitats, respectively. n=Population.SpeciesCount is the number of species in the current island habitat, and P=OPTIONS.popsize is the maximum number of species.

**Figure 6:**
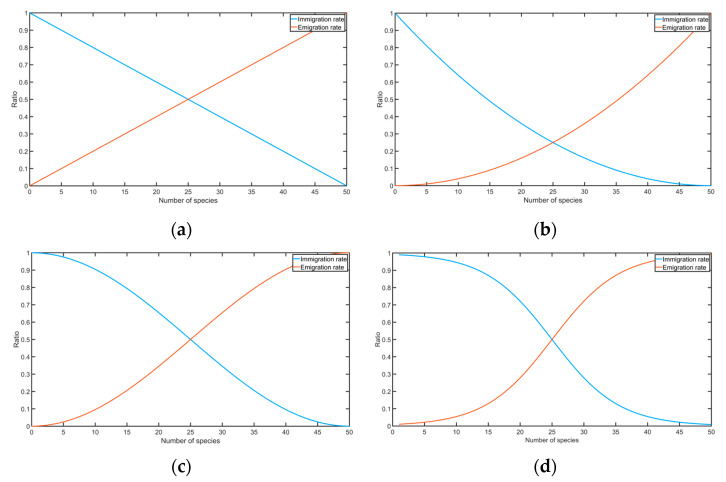
(**a**) Transition plot for a linear function model; (**b**) transition plot for quadratic function model; (**c**) transition plot for the cosine function model; (**d**) transition plot for hyperbolic tangent function model.

In this hyperbolic tangent mobility model (α was taken as 1.1 in this paper), the trend of mobility changing with the number of species is similar to that of the cosine model, but the amplitude is weaker than that of the cosine model when the number of species is small. The amplitude is stronger in the middle, which better describes the actual law of species migration between island habitats. Several transfer models are shown in Figure 6 (I=E=50).

#### 3.3.3. Improvement of Migration Operator

In Simon’s BBO algorithm, when the random number generated by a certain dimension is smaller than the emigration rate, the migration operator will directly replace the corresponding features of the immigration island habitat with the characteristics of the immigration island habitat; when the random number is greater than the emigration island habitat rate, no change is made. This may allow the characteristics of poor individuals to be replicated in better individuals. This paper proposes the following improved algorithms:

- Weight transfer operator. When the random number of a certain dimension is less than the emigration rate, 10% of the original island habitat disturbance is added, and the weight of the emigrated island habitat is reduced to 90%, i.e.,
  (13) $$\begin{array}{l} Island(k,j)=0.1\times Populatin(k).chrom(j)+\\ 0.9\times Population(SelectIndex).chrom(j) \end{array}$$
  where Island(k,j) is the j-th dimension vector in the k-th island habitat after the migration operation is completed, Populatin(k).chrom(j) is the j-component of the original island habitat k, and Population(SelectIndex).chrom(j) is the j-component of the SelectIndex-th island habitat selected to provide exchange information.

- Mixed optimal migration operator. When the random number of a certain dimension is greater than the migration rate, the variable of this dimension is still migrated, and the migration method is the convex combination of the migrated individual and the current optimal individual p1, i.e.,
  (14) $$\begin{array}{l} Island(k,j)=(1-\varepsilon )\times Population(k).chrom(j)+\\ \varepsilon \times Population(1).chrom(j) \end{array}$$
  where ε∈[0,1], and Population(1).chrom(j) is the j-th dimension vector of the current best individual.

The reasons for using mixed transfer are that good individuals are less likely to degenerate due to transfer, because some of their original characteristics will be retained during the transfer process; poor individuals will receive at least part of the solution from good individuals during transfer. Features such as migration ensure that species evolve towards the optimal value of each generation, no longer blindly searching, and they can quickly converge toward the optimal direction.

The mixing parameter ε can be random, deterministic, or dynamically changed. In this paper, a strategy of dynamically adjusting q according to the change of the number of iterations is proposed through a large number of experiments. In order to for the island habitat to be mainly affected by the characteristics of the immigrant island habitat in the early stage of evolution, the mixing parameter $\varepsilon_{1}=0.1$ is taken. In the middle stage of evolution, in order to prevent the algorithm from prematurely falling into the local optimum, while ensuring that individuals with low fitness have the ability to survive and develop, the mixed migration parameter $\varepsilon_{2}=0.5$ is taken to reduce the migration pressure. In the later stage of evolution, in order to reduce the random disturbance of migrating island habitats and destroy the better individuals, the mixing parameter $\varepsilon_{3}=0.9$ is taken to make the better solution have stronger survivability, which helps to improve the convergence accuracy. The entire migration equation is as follows:

(15) $$\begin{array}{l} \mathrm{for}\;\mathrm{each}\;\mathrm{individual}\\ \;\;\mathrm{for}\;\mathrm{each}\;\mathrm{dimension}\\ \;\;\;\mathrm{if}\;rand<lambdaScale\\ \;\;\;Island(k,j)=0.1\times Population(k).chrom(j)+0.9\times Population(SelectIndex).chrom(j)\\ \;\;\;\mathrm{else}\\ \;\;\;\;\;\mathrm{if}\;GenIndex<OPTION.Maxgen/3\\ \;\;\;\;\;Island(k,j)=(1-\varepsilon_{1})\times Population(k).chrom(j)+\varepsilon_{1}\times Population(1).chrom(j)\;\varepsilon_{1}=0.1\\ \;\;\;\;\mathrm{else}\;\\ \;\;\;\;\;\;\;\mathrm{if}\;OPTION.Maxgen/3<GenIndex<=2\times OPTION.Maxgen/3\\ \;\;\;\;\;\;\;Island(k,j)=(1-\varepsilon_{2})\times Population(k).chrom(j)+\varepsilon_{2}\times Population(1).chrom(j)\;\varepsilon_{2}=0.5\\ \;\;\;\;\;\;\mathrm{else}\;\\ \;\;\;\;\;\;\;\;\;Island(k,j)=(1-\varepsilon_{3})\times Population(k).chrom(j)+\varepsilon_{3}\times Population(1).chrom(j)\;\varepsilon_{3}=0.9\\ \;\;\;\;\;\;\;\;\mathrm{end}\;\mathrm{if}\\ \;\;\;\;\;\;\mathrm{end}\;\mathrm{if}\\ \;\;\;\;\mathrm{end}\;\mathrm{if}\\ \;\;\;\mathrm{next}\;\mathrm{dimension}\\ \;\mathrm{next}\;\mathrm{individual} \end{array}$$

where lambdaScale (normalized immigration rate) is the standard immigration rate, GenIndex is the current number of iterations, and OPTIONS.Maxgen is the maximum number of iterations.

#### 3.3.4. Improvement of Mutation Operator

The standard BBO adopts a random mutation strategy, which facilitates destroying individuals with high fitness, resulting in the mutation potentially bringing about worse individuals and reducing diversity. This paper proposes an optimal hybrid mutation operator to avoid the drawbacks caused by random mutation. The specific algorithm implementation is divided into two parts, optimization and mixing, with weights of 0.618 and 0.382, respectively, after many tests.

The specific operation is to multiply the optimal value component Population(1).chrom(parnum) obtained in the current island habitat (the parnum-th dimension component in the first island habitat) by the weight 0.628 as the optimal component; the p-th dimensional component of the k-th island habitat is multiplied by 0.328 and then multiplied by the random number of variation as a mixed component, which is embodied in the student distribution (t distribution) trand(1,1,1) in the early stage of the iteration to obtain the large-scale variable asynchronous length. In the later stage of the iteration, the main purpose of the Gaussian variation normrand(0,1) is to strengthen the local exploration ability in the later stage and jump out of the limitation of local optimization. The entire mutation algorithm is as follows:

(16) $$\begin{array}{l} Island(k,parnum)=0.618\times Population(1).chrom(parnum)+\\ 0.328\times Populatin(k).chrom(parnum)\times \\ (GenIndex/OPTION.Maxgen\times (1+normrnd(0,1))+\\ \left(1-GenIndex/OPTIONS.Maxgen)\times (1+trand(1,1,1))\right) \end{array}$$

### 3.4. Simulation Experiment and Result Analysis

In order to verify and test the practicability and advancement of the improved BBO algorithm in this paper, a series of comparative experiments are carried out in this section, mainly to compare the improved BBO algorithm with the original BBO algorithm and various excellent intelligent optimization algorithms in recent years, including ACO, DE, ES, GA, PBIL, PSO, and SGA. This article uses 13 standard benchmark functions as shown in Table 1.

Nine algorithms were tested on these 13 test functions, with the species scale OPTION.popsize=50 and the maximum number of iterations OPTION.Maxgen=200. The maximum immigrant rate I and the maximum immigrant rate E were both 1, the maximum number of species $S_{\max}=P=OPTION.popsize$, and the maximum mutation rate $m_{\max}=OPTION.pmutate=0.005$. In order to avoid chance and maintain the scientificity and rigor of the experiment, the nine algorithms were independently run 50 times on each test function, and the mean and standard deviation of the 50 results were compared. The comparison results of the nine algorithms are shown in Table 2.

**Figure 7:**
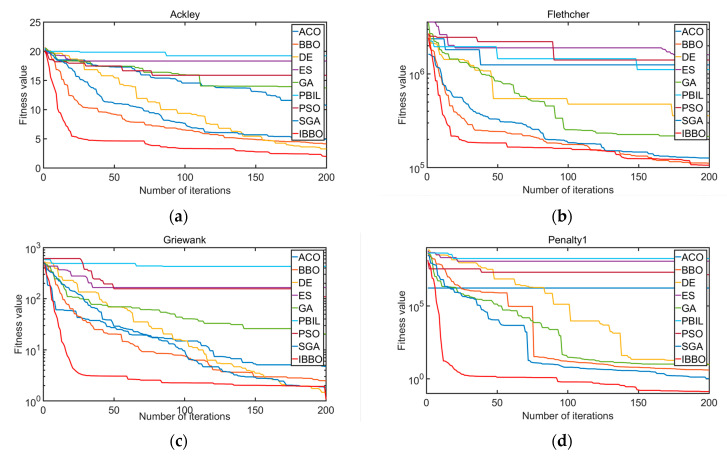

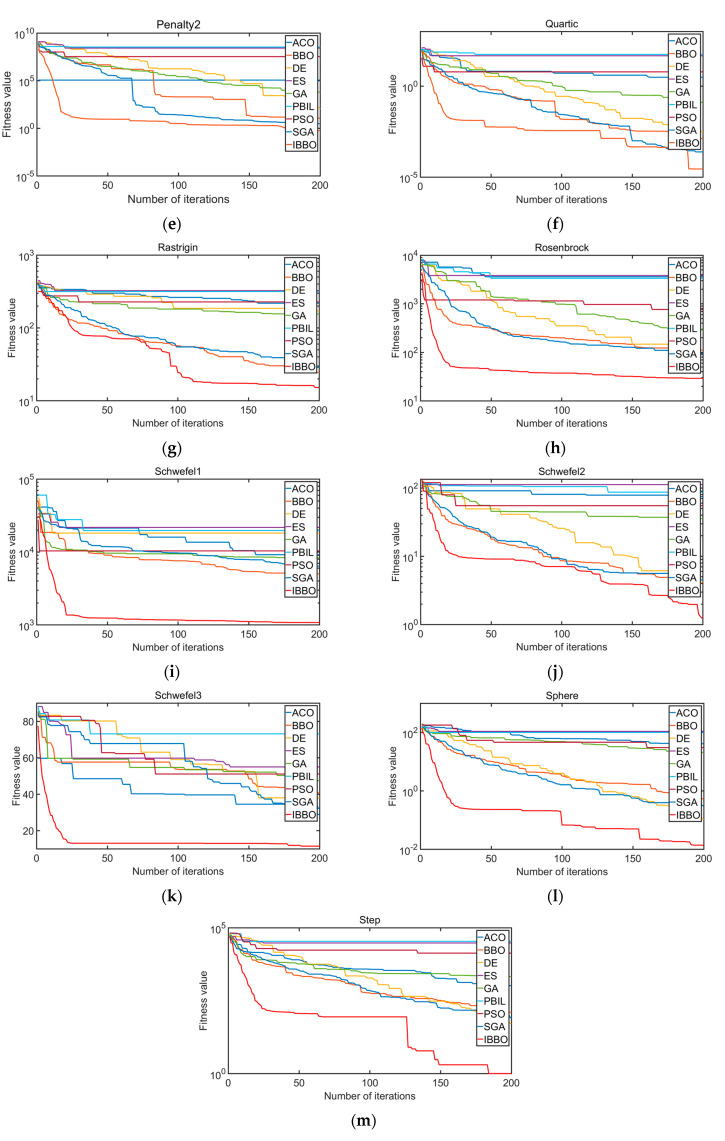
(**a**) Convergence of nine algorithms under Ackley function; (**b**) convergence of nine algorithms under Flethcher function; (**c**) convergence of nine algorithms under Griewank function; (**d**) convergence of nine algorithms under Penalty1 function; (**e**) convergence of nine algorithms under Penalty2 function; (**f**) convergence of nine algorithms under Quartic function; (**g**) convergence of nine algorithms under Rastrigin function; (**h**) convergence of nine algorithms under Rosenbrock function; (**i**) convergence of nine algorithms under Schwefel1 function; (**j**) convergence of nine algorithms under Schwefel2 function; (**k**) convergence of nine algorithms under Schwefel3 function; (**l**) convergence of nine algorithms under Sphere function; (**m**) convergence of nine algorithms under Step function.

In addition, the convergence of the nine algorithms to the 13 test functions is shown in Figure 7.

It can be seen from Table 2 and Figure 7 that, compared with the other eight algorithms, IBBO had a good performance in the selected 13 test functions. Specifically, the minimum value, the mean value, and the standard deviation were relatively smaller, and the convergence speed and accuracy were also relatively better. This shows that the IBBO algorithm had better global search ability and the ability to jump out of the local optimum, representing an improved algorithm worthy of adoption and promotion.

## 4. Parameter Identification of Pneumatic Control Valve Model

**Figure 8:**
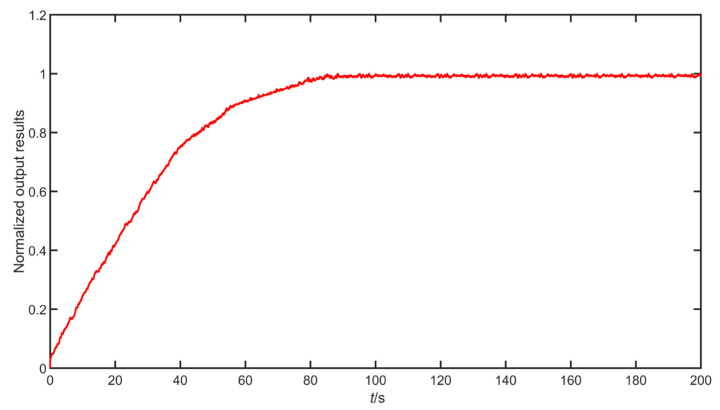
Open-loop step normalization results of pneumatic control valve.

The pneumatic control valve integrates the air circuit, circuit, and magnetic circuit, and the control system is complex. The white box model often does not take into account the viscosity and wear of the control valve, and it requires a large number of accurate parameters; when the model deviates from the actual process, the actual process data also need to be compensated. An open-loop step experiment was performed on the pneumatic control valve, and the results were normalized. The experimental results are shown in Figure 8.

**Figure 9:**
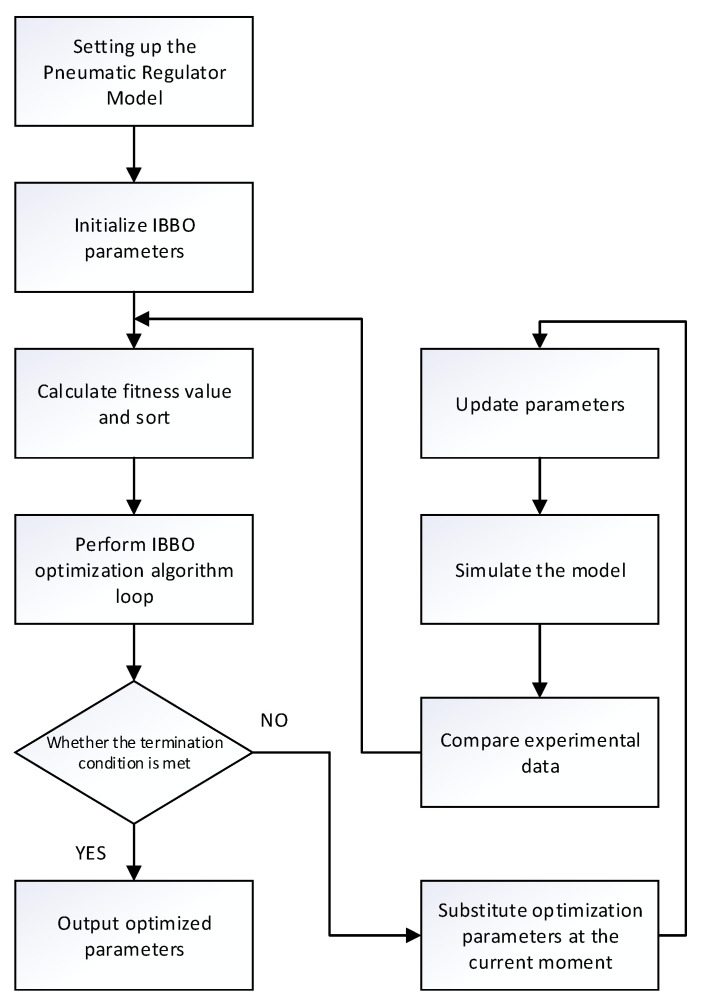
Improved biogeography-based optimization algorithm identification model flow chart.

The pneumatic control valve model is often expressed in the form of a third-order transfer function [23]. In order to establish an effective model, the transfer function was identified through the improved biogeography-based optimization algorithm in this paper, and the step response of the model was compared with the experimental data at each iteration. The difference was used as the algorithm adaptation value. The flow chart is shown in Figure 9.

After many iterations, the pneumatic control valve model was obtained as follows:

(17) $$G(s)=\frac{5.588}{12.370S^{3}+620.851S^{2}+164.916S+5.568}e^{-0.32}.$$

**Figure 10:**
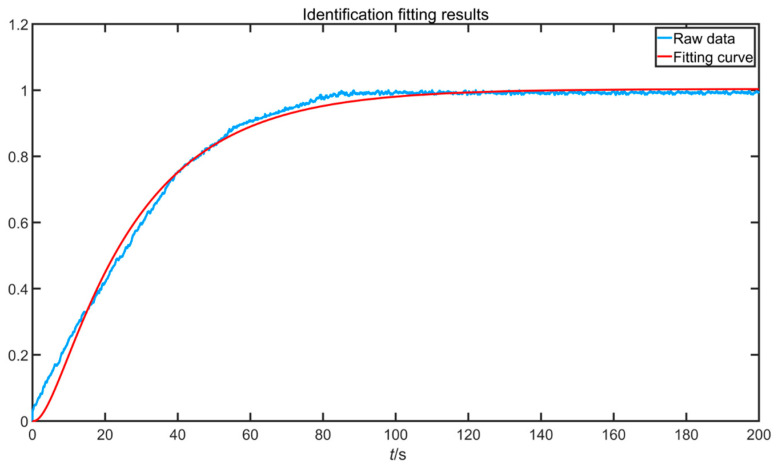
Identify fit plots.

Its identification fitting diagram is shown in Figure 10.

It can be seen from Figure 10 that the proposed improved biogeography optimization algorithm had a high degree of fitting, which shows the effectiveness and superiority of the improved algorithm.

## 5. Fractional-Order PID Controller Design

### 5.1. Fractional-Order PID Controller

There are three commonly used definitions of fractional calculus: Riemann−Liouville, Grunwald−Letnikov, and Caputo definitions. Under certain conditions, the first two definitions are basically equivalent, where Grunwald−Letnikov is defined as

**Figure 11:**
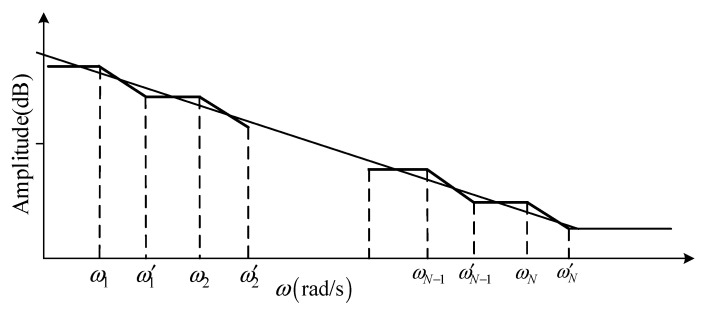
Piecewise polyline approximation of filters.

(18) Dtαt0f(t)=limh→01hα∑j=0[t−t0h](−1)j(αj)f(t−jh),

where $\left(\begin{array}{c} \alpha \\ j \end{array}\right)=\frac{\Gamma \left(\alpha +1\right)}{\Gamma \left(j+1\right)\Gamma \left(\alpha -j+1\right)}$, [·] is the nearest integer, α is the calculus order, $t_{0}$ and t are the upper and lower limits of the integral, respectively, and h is the sampling period. The discrete and approximate fractional operators are realized using the Oustaloup filter. If the frequency band of the approximate model to be obtained is $\left[w_{b},w_{h}\right]$, the linear characteristics of fractional calculus can be approximated according to a group of broken lines, as shown in the Figure 11.

**Figure 12:**
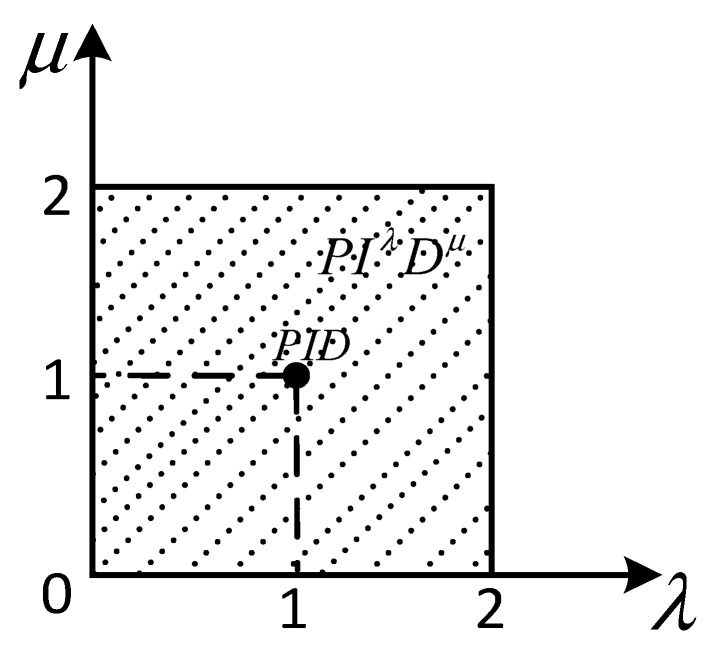
Fractional-order PID control plane diagram.

Thus, the transfer function is

(19) $$G=K\overset{N}{\underset{k=-N}{\prod }}\frac{1+\frac{s}{{w^{\prime }}_{k}}}{1+\frac{s}{w_{k}}},$$

where K is the gain, N is the order, and ${w^{\prime }}_{k}$ and $w_{k}$ represent the zero and pole respectively.

(20) $$K={\left(w_{h},w_{b}\right)}^{\delta },$$

(21) $${w^{\prime }}_{k}=w_{b}{\left(\frac{w_{h}}{w_{b}}\right)}^{\frac{k+N+0.5\left(1-\mu \right)}{2N+1}},$$

(22) $$w_{k}=w_{b}{\left(\frac{w_{h}}{w_{b}}\right)}^{\frac{k+N+0.5\left(1+\mu \right)}{2N+1}},$$

where δ is the fractional order, $w_{b}=0.001$, $w_{h}=10000$, and N=5. Compared with integer-order PID, FOPID has more integral order λ and differential order μ, which can control the controlled object more flexibly, so as to meet the performance index of complex system. Figure 12 is the FOPID control plane diagram.

**Figure 13:**
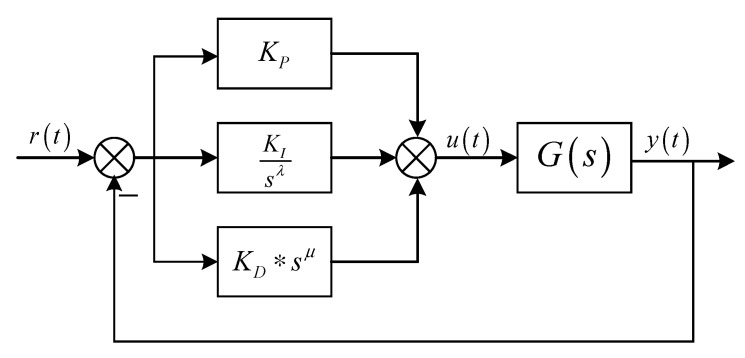
Fractional-order PID control system model.

Figure 13 is the FOPID control system model. r(t), u(t), and y(t) are the expected input, controller output, and system output, respectively, and G(s) is the controlled object.

The transfer function of the fractional-order PID controller is as follows:

(23) $$G\left(s\right)=K_{P}+\frac{K_{I}}{s^{\lambda }}+K_{D}s^{\mu }.$$

### 5.2. Parameter Tuning of Fractional-Order PID Controller

In this paper, according to the design performance index of the valve opening output signal of the valve control system, $K_{p}$, $K_{i}$, $K_{d}$, λ, and μ are regarded as five components of a single particle, and the optimization calculation is carried out in the five-dimensional space.

The value of the fitness function is used to judge the quality of the parameter optimization result, and the ITAE performance index (the absolute value error of the system and the integral of time) can be selected to reflect the accuracy and rapidity of the system, while taking into account the small overshoot. The square term of the controller output is added to the fitness function to avoid excessive output. The fitness function is

(24) $$J=\overset{T}{\underset{0}{\int }}\left(\lambda_{1}t\left|e\left(t\right)\right|+\lambda_{2}u^{2}\left(t\right)\right)dt,$$

where e(t) is the control error, u(t) is the output of the controller, and $\lambda_{1}$ and $\lambda_{2}$ are the weight coefficients ($\lambda_{1}$ and $\lambda_{2}$ are 0.999 and 0.001, respectively). The steps of improving the biogeographic optimization algorithm to optimize fractional PID parameters are described below.

Step 1: Initialize the island habitat scale OPTIONS.popsize, the optimization dimension OPTIONS.numVar, the maximum number of iterations OPTIONS.Maxgen, the chaos variable μ, the elite retention rate Keep, the initial mutation rate OPTIONS.pmutate, etc., and define the upper and lower limits of each dimension.

Step 2: Evaluate the HSI of each island habitat and rank the species according to the HSI from good to bad.

Step 3: Calculate the in-, out-, and mutation rates for each island habitat, preserving elite island habitats.

Step 4: Execute the weight transfer operator and the hybrid convergence transfer operator.

Step 5: Execute the optimal hybrid mutation operator.

Step 6: Make out-of-bounds restrictions for each island habitat.

Step 7: Evaluate HSI for each island habitat, rank species according to HSI from good to bad, and replace poor island habitat with elite island habitat.

Step 8: Sort species according to HSI from good to bad.

Step 9: Judge whether the maximum number of iterations or the search accuracy requirement is met; if neither is satisfied, the number of iterations is increased by one, and the process returns to Step 3 until the termination condition is reached.

### 5.3. Simulation

In order to test the effect of IBBO optimizing FOPID parameters, the transfer function of Equation (17) was used, and the control model was built with Simulink. The standard BBO, IBBO, and SGA algorithms (the SGA algorithm has certain advantages in comparison of test functions compared to BBO-related algorithms) were set with species scale A = 50, maximum number of iterations M = 100, FOPID parameter value range $K_{p}\in \left[0.001,300\right]$, $K_{i}\in \left[0.001,300\right]$, $K_{d}\in \left[0.001,300\right]$, λ∈[0.001,2], and μ∈[0.001,2]; the given system input was a unit step signal. The standard BBO algorithm, IPPO algorithm, and SGA algorithm were each carried out for 20 experiments, and the optimal value was taken as the parameter comparison. The fitness value curve is shown below.

**Figure 14:**
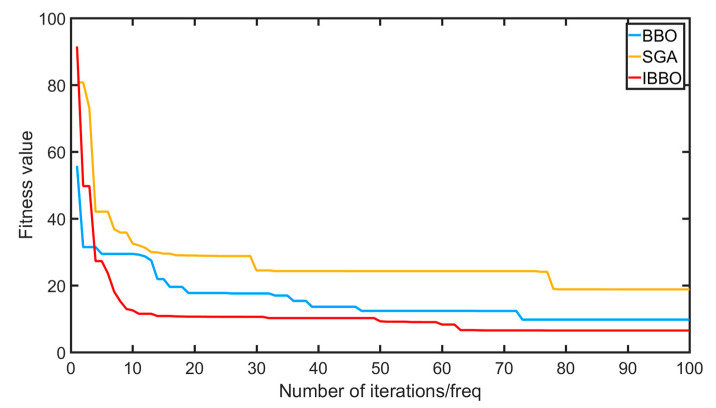
Optimal individual fitness value changes of three algorithms.

As shown in Figure 14, compared with the standard BBO and SGA algorithms, the optimal fitness value of the IBBO algorithm has a faster convergence speed and can better jump out of the local optimal value, i.e., the found parameters are better; hence, the IBBO has a higher search rate, accuracy, and convergence speed.

**Figure 15:**
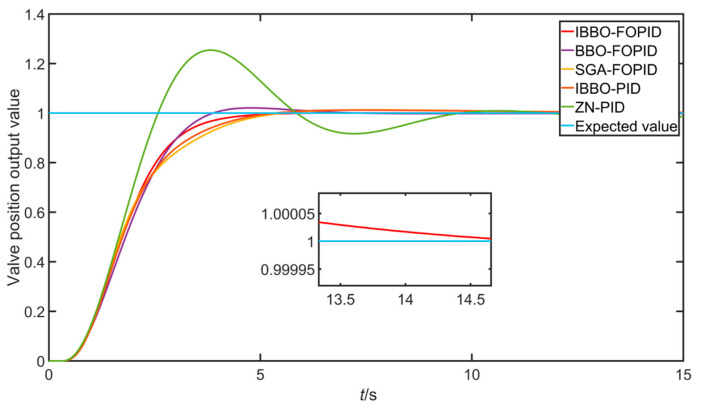
Comparison of step responses of different algorithms.

The parameter values optimized by the three methods were used for the simulation experiment of valve opening control, the control parameters under the optimal fitness value after optimization were taken, and the simulation experiment was carried out under the unit step input. The results are shown in Figure 15.

The three performance indicators of overshoot, adjustment time (calculated by 5%), and steady-state error of the five algorithms were compared, and the results are shown in Table 3.

As can be seen from Figure 15 and Table 3, compared with the other four optimized algorithms, the IBBO optimization algorithm had the advantages of small overshoot, short adjustment time, and low steady-state error. In addition, it can be seen from Table 3 that, under the parameter optimization of the same algorithm, compared with the integer-order PID, the FOPID had a smaller steady-state error, reduced overshoot, and a better dynamic performance.

**Figure 16:**
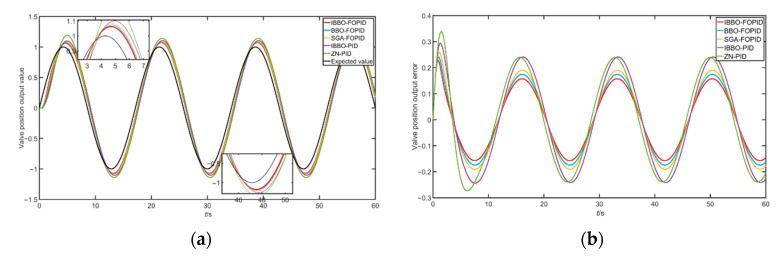
(**a**) Tracking curve of sinusoidal signal; (**b**) tracking error of sinusoidal signal.

In order to further verify the performance of the control algorithm, a set of sinusoidal signals $y=\sin\left(\frac{7\pi }{60}t\right)$ were set as the desired valve position opening signal of the simulation system. Figure 16 shows the tracking of the target valve position by the valve position opening control system under the action of five different algorithms. From the control system effect, it can be concluded that the IBBO optimized algorithm had a better effect on the tracking control of the valve position of the regulating valve.

## 6. Experimental Verification

**Figure 17:**
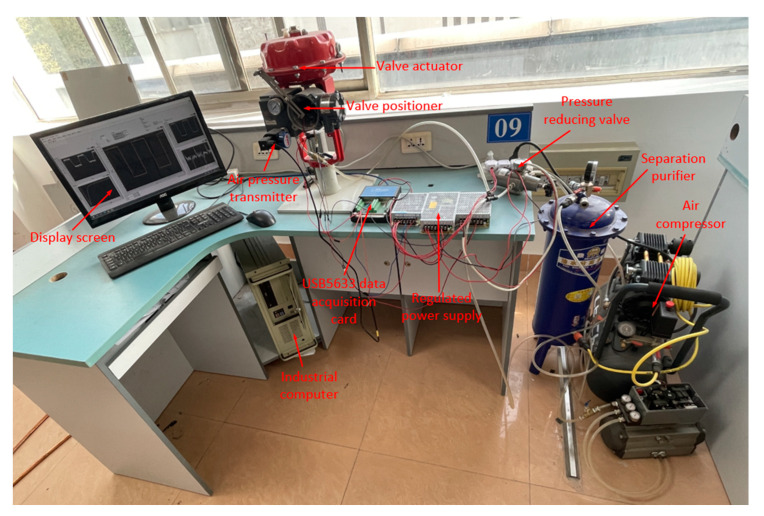
Experimental equipment and devices.

In order to test the effectiveness of the fractional-order PID controller, an experimental platform for pneumatic control valves was established, and two different controller algorithms were written through the LabVIEW graphical programming software on the upper computer. The equipment of the experimental platform is shown in Figure 17.

The working pressure of the pneumatic control valve was set to 0.6 MPa, the feedback voltage signal of the valve positioner was collected by USB5633, and the voltage signal in the range of 0–5 V was output to drive the movement of the control valve stem.

The step, sine wave, and square wave signals were used as the output expected valve position, and the integer-order PID controller and the fractional-order PID controller were used to track the set expected valve position value.

- The output experiment of a given step signal mainly tests the transient performance of the system. The desired signal of the valve position opening with a given output of 50% and the experimental results of air pressure are shown below.

**Figure 18:**
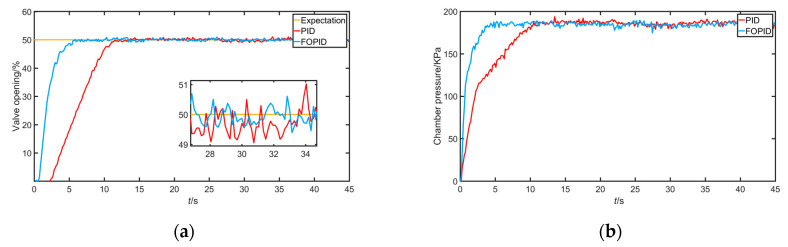
(**a**) Response signal under 50% valve position opening tracking; (**b**) error of response signal under 50% valve position opening tracking.

According to Figure 18 and Table 4, in terms of transient response, the overshoot of PID and FOPID was almost 0, whereby FOPID was slightly better than integer-order PID. In terms of rise time and adjustment time, FOPID was better. It can also be seen that the transient performance of the FOPID controller was better than that of the PID controller. In terms of steady state, compared with PID, the FOPID controller reduced the system steady-state error and increased the control accuracy.

**Figure 19:**
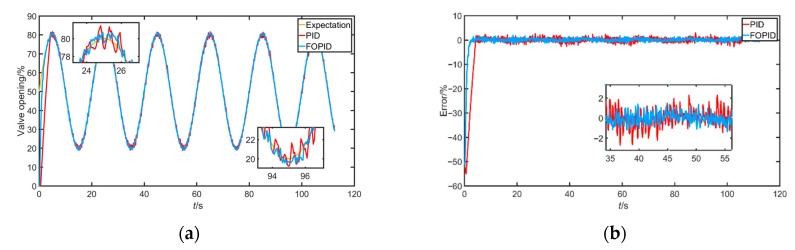

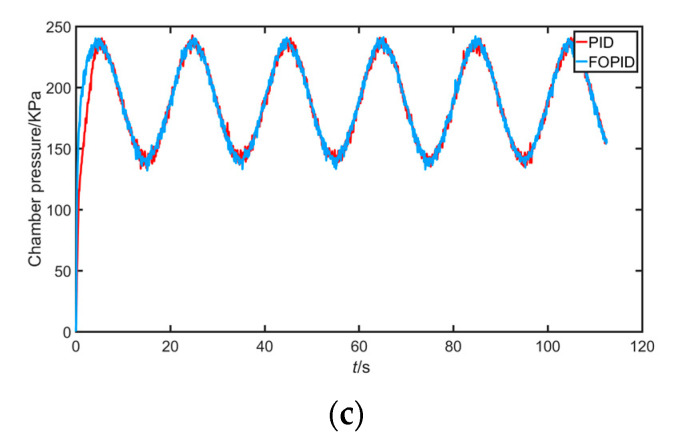
(**a**) Response signal under sinusoidal tracking; (**b**) error of response signal under sinusoidal tracking; (**c**) corresponding air chamber pressure under sinusoidal signal.

- The output experiment of a given sine wave mainly tests the dynamic performance of the controller. In the experiment, the desired valve position signal was selected as $y=30*\sin\left(\frac{\pi }{10}t\right)+50$. The experimental results are shown below in Figure 19.

It can be clearly seen from the sinusoidal tracking curve and error curve that FOPID could track the expected input more quickly and had a smaller oscillation curve (smaller tracking error), indicating greater advantages of FOPID in dynamic performance than PID.

- Given the square wave output experiment, the main purpose is to test the controller’s fast performance and its ability to track mutation signals. The valve position expected value output was set to 80%–20%–80%, i.e., $y=30*square\left(\frac{\pi }{10}t\right)+50$, and the experiment repeated four to five times. The results are shown below.

**Figure 20:**
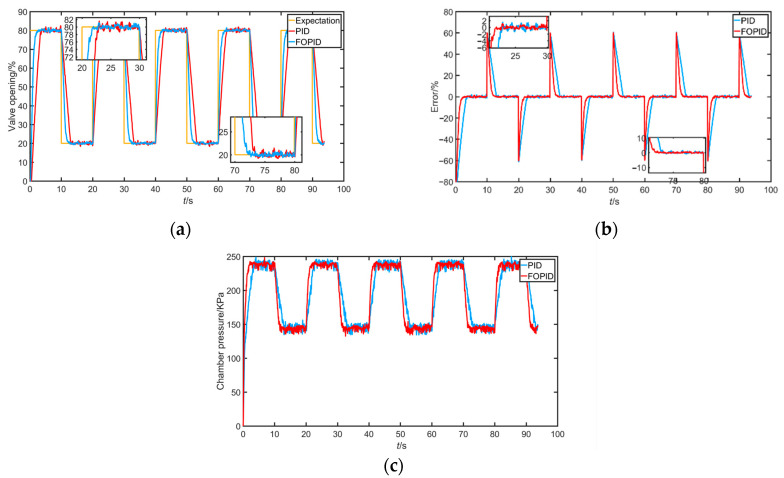
(**a**) Response signal under square wave tracking; (**b**) error of response signal under square wave tracking; (**c**) corresponding air chamber pressure under square wave tracking.

It can be seen from Figure 20 that, when the given signal was abruptly changed in the forward or reverse direction, the system under FOPID control could track the given signal more quickly and almost without overshoot, and the tracking error was smaller. Obviously, the FOPID controller had better dynamic performance.

At the same time, in order to further evaluate the performance of the two methods, the root-mean-square error (*RMSE*) and the mean absolute percentage error (*MAPE*) were introduced for further comparison and explanation, which are defined as follows:

(25) $$RMSE=\sqrt{\frac{1}{n}\sum_{i=1}^{n}{\left({\hat{y}}_{i}-y_{i}\right)}^{2}},$$

(26) $$MAPE=\frac{100\%}{n}\sum_{i=1}^{n}\left|\frac{{\hat{y}}_{i}-y_{i}}{{\hat{y}}_{i}}\right|,$$

where n is the number of sampling points, and ${\overset{⌢}{y}}_{i}$ and $y_{i}$ are the predicted value and actual value of the i-th point, respectively.

According to the comparison of the indicators in Table 5, the FOPID controller had a smaller root-mean-square error and average absolute percentage error for control of the pneumatic control valve position, and the control accuracy of the control valve was better than that of the PID controller.

The experimental and simulation data are shown in Table 6. It can be seen from the table that the control effect of the FOPID algorithm was better than that of the corresponding PID algorithm in the comparison of overshoot and adjustment time.

## 7. Conclusions

In this paper, we first proposed an IBBO algorithm, which can improve the optimization ability by introducing chaotic map initialization, adjusting the migration model, and improving the migration and mutation operators. This algorithm is not reflected in the current literature. In the simulation comparison of 13 test functions, the minimum value, mean value, and standard deviation of the IBBO algorithm were relatively smaller, and the convergence speed and accuracy were also relatively better. Taking the sphere test function as an example, its minimum value, mean value, and standard deviation were increased by 87.0%, 77.9%, and 62.6%, respectively. Then, on the basis of the proposed IBBO algorithm, the open-loop response curve of the control system was fitted, and the model parameters of the pneumatic control valve were identified. Then, the FOPID control algorithm was introduced, and the parameters of the FOPID controller were adjusted by the IBBO algorithm to realize the control of the pneumatic control valve position. This control method is not reflected in the current literature. Lastly, through simulation, the overshoot and steady-state error of the IBBO-FOPID control algorithm were only 0.6760 and 0.0008, increasing by 45.6% and 42.8%, respectively. According to the experimental verification, FOPID was better than PID, proving the effectiveness of the proposed control method for regulating valve position.

## Figures and Tables

**Table 1:** The 13 standard benchmark functions.

Function Name	Number of Dimensions	Scope
Ackley	30	${\left[-30,30\right]}^{30}$
Flethcher	30	${\left[-\pi ,\pi \right]}^{30}$
Griewank	30	${\left[-600,600\right]}^{30}$
Penalty1	30	${\left[-50,50\right]}^{30}$
Penalty2	30	${\left[-50,50\right]}^{30}$
Quartic	30	${\left[-1.28,1.28\right]}^{30}$
Rastrigin	30	${\left[-5.15,5.12\right]}^{30}$
Rosenbrock	30	${\left[-2.048,2.048\right]}^{{}^{30}}$
Schwefel1	30	${\left[-65.536,65.536\right]}^{30}$
Schwefel2	30	${\left[-20,20\right]}^{30}$
Schwefel3	30	${\left[-200,200\right]}^{30}$
Sphere	30	${\left[-5.12,5.12\right]}^{30}$
Step	30	${\left[-200,200\right]}^{30}$

**Table 2:** Comparison results of nine algorithms for 13 test functions.

Function		ACO	BBO	DE	ES	GA	PBIL	PSO	SGA	IBBO
Ackley	min	10.6628	4.1256	3.2636	18.3172	13.7105	19.2149	15.8786	3.9221	1.9716
	mean	13.5510	4.8965	3.6056	19.3152	16.7410	19.8378	16.3563	4.6009	2.4420
	std	2.3223	0.5765	0.2466	0.3891	1.4076	0.246	0.3399	0.5236	0.2927
Flenthcher	min	1,245,598	111,062	356,329	1,597,417	214,239	1,109,620	1,403,971	125,131	105,709
	mean	1.8 × 10^6^	1.3 × 10^5^	4.8 × 10^5^	1.9 × 10^6^	4.2 × 10^5^	1.4 × 10^6^	1.6 × 10^6^	1.6 × 10^5^	1.3 × 10^5^
	std	4.0 × 10^5^	2.2 × 10^4^	6.6 × 10^4^	2.4 × 10^5^	1.5 × 10^5^	2.0× 10^5^	1.5 × 10^5^	2.3 × 10^4^	1.1 × 10^4^
Griewank	min	4.7090	2.4367	1.4684	165.044	20.2711	427.8509	108.994	1.8198	1.0679
	mean	7.2543	2.9381	1.6811	221.703	37.9814	448.9497	140.198	2.2808	1.2268
	std	1.6275	0.3260	0.2198	33.3833	10.8546	24.1245	19.3807	0.5274	0.0677
Penalty1	min	1,671,844	3.9859	8.6215	1.2 × 10^8^	9.4521	1.8 × 10^8^	13,750,807	1.0272	0.1321
	mean	3.4 × 10^8^	4.5284	14.1119	1.8 × 10^8^	864.382	2.7 × 10^8^	2.0 × 10^7^	1.4334	0.4603
	std	2.7 × 10^8^	0.5636	3.7758	5.1 × 10^7^	864.380	8.0 × 10^7^	7.0 × 10^6^	0.5340	0.1815
Penalty2	min	113,520	11.4652	105.1962	2.6 × 10^8^	6259.28	3.3 × 10^8^	3.3 × 10^7^	3.0526	0.5626
	mean	3.0 × 10^8^	17.8096	739.8444	3.7 × 10^8^	3.0 × 10^5^	5.9 × 10^8^	7.3 × 10^7^	4.6854	2.5750
	std	3.5 × 10^8^	5.9365	686.6578	6.1 × 10^7^	4.0 × 10^5^	1.0 × 10^8^	3.2 × 10^7^	1.2751	1.0279
Quartic	min	3.0487	0.0013	0.0031	46.1170	0.1269	54.7615	6.2594	0.0002	2.8 × 10^−5^
	mean	5.8396	0.0053	0.0067	58.5713	0.6576	65.9201	8.6888	3.9 × 10^−4^	5.7 × 10^−5^
	std	3.6090	0.0040	0.0033	7.3838	0.3701	5.9463	2.5195	1.2 × 10^−4^	2.4 × 10^−5^
Rastrigin	min	217.8773	24.3133	169.2863	323.992	152.970	315.287	226.959	28.6717	15.1721
	mean	251.5205	27.9260	186.6605	366.190	203.196	364.956	260.566	34.9787	19.6687
	std	20.4680	3.1046	7.7485	23.5157	36.4538	18.6455	17.4848	3.8464	1.9322
Rosenbrock	min	3594.80	111.203	94.562	3816.12	288.426	3349.00	763.314	94.9775	29.5112
	mean	4.4 × 10^3^	132.272	108.888	5.1 × 10^3^	405.606	4.4 × 10^3^	1.2 × 10^3^	115.377	33.7508
	std	451.691	22.5807	11.1501	926.611	91.3460	571.998	237.121	17.9313	2.7769
Schwefel1	min	9162.77	5002.61	18,129.05	21,679.1	8277.50	19,694.6	10,323.2	6388.35	1074.32
	mean	1.4 × 10^4^	7.0 × 10^3^	2.1 × 10^4^	2.9 × 10^4^	1.5 × 10^4^	2.4 × 10^4^	1.7 × 10^4^	9.4 × 10^3^	1.2 × 10^3^
	std	2.8 × 10^3^	902.9220	2.4 × 10^3^	4.1 × 10^3^	3.8 × 10^3^	2.7 × 10^3^	3.2 × 10^3^	2.2 × 10^3^	108.255
Schwefel2	min	74.8000	4.1000	4.1630	111.900	35.6000	86.7000	54.9496	4.5000	1.2521
	mean	84.3500	4.8800	4.6133	125.570	46.9900	100.110	102.771	6.5200	2.1421
	std	6.4977	0.6431	0.4821	10.6046	6.9542	5.9831	31.3971	1.3578	0.3756
Schwefel3	min	32.2000	40.7000	37.9842	54.9440	47.4000	73.1000	51.0582	28.9000	11.4676
	mean	38.5400	49.4200	45.4736	61.9637	55.4400	77.3600	60.8716	39.7100	14.6438
	std	5.4244	6.8949	5.1956	5.1824	6.8050	2.2962	8.5732	6.8140	1.2038
Sphere	min	40.9896	0.5241	0.1052	108.830	20.6913	100.080	30.23603	0.2923	0.0137
	mean	50.8767	0.6823	0.1689	132.356	31.2184	128.365	38.4796	0.4767	0.0374
	std	6.7235	0.0974	0.0439	13.5977	6.7534	13.1963	4.2327	0.1093	0.0164
Step	min	1036.00	127.000	53.0000	30,114.0	2126.00	34,324.0	13,524.0	80.0000	1.0000
	mean	1.5 × 10^3^	206.900	69.0000	34,528.0	3.5 × 10^3^	46,144.0	15,917.0	122.600	10.8000
	std	360.716	53.2531	9.5812	4.0 × 10^3^	1.3 × 10^3^	6.2 × 10^3^	1.5 × 10^3^	52.8076	4.6648

**Table 3:** Comparison of performance indicators of five algorithms.

Performance Indicators	IBBO-FOPID	BBO-FOPID	SGA-FOPID	IBBO-PID	ZN-PID
Overshoot (%)	0.6760	2.0967	1.2869	1.2440	25.4546
Adjustment time (s)	3.5343	3.3502	4.1814	4.0370	8.4253
Steady-state error	0.0008	0.0014	0.0018	0.0025	0.0142

**Table 4:** The corresponding performance indicators of the two algorithms at 50% opening.

Performance Indicators	Overshoot (%)	Rise Time (s)	Adjustment Time (s)
PID	0.010207	6.45	10.35
FOPID	0.009575	2.55	4.50

**Table 5:** Comparison of performance indicators between PID and FOPID.

Performance Indicators	RMSE		MAPE (%)	
	Sin Signal	Square Signal	Sin Signal	Square Signal
PID	6.8681	20.7357	3.4602	32.2391
FOPID	3.6245	14.0825	1.7184	16.2357

**Table 6:** Comparison of PID algorithm and FOPID algorithm in 50% opening step input simulation and experiment.

Performance Indicators	PID		FOPID	
	Simulation	Experiment	Simulation	Experiment
Overshoot (%)	1.2440	0.0102	0.6760	0.0095
Adjustment time (s)	4.0370	10.3500	3.5343	4.5000

## Data Availability

Not applicable.
